# Treatment of type II symptomatic ulnar styloid nonunions with reinsertion of the triangular fibrocartilage complex

**DOI:** 10.1186/s12891-023-06718-x

**Published:** 2023-08-09

**Authors:** Xiaofei Yu, Yadong Yu, Xu Zhang, Jia Li, Tong Zhou, Huan Chen

**Affiliations:** 1 Department of Hand Surgery, Third Hospital of Heibei Medical University, Shijiazhuang, 050051 Hebei China; 2 The Second Hospital of Tangshan, Hebei, China; 3 The Second Affiliated Hospital of Wannan Medical College, Hebei, China

**Keywords:** Ulnar styloid fracture, Nonunion, Ulnar wrist pain, Distal radioulnar joint

## Abstract

**Purpose:**

The purpose of this retrospective study was to introduce an alternative technique for the treatment of type II symptomatic ulnar styloid nonunion by the reinsertion of the triangular fibrocartilage complex and the ulnar collateral ligament.

**Methods:**

Between March 2009 and May 2017, 45 patients (34 males and 11 females) suffering from the nonunion of type II ulnar styloid fractures all underwent the subperiosteal resection of the avulsed fragments and the reinsertion of the TFCC and ulnar collateral ligament. Outcome assessments included the ranges of motion of the wrist, grip strength, pain, and Mayo wrist score. The preoperative and postoperative parameters were compared. A P-value less than 0.05 was considered to be statistically significant.

**Result:**

The mean follow-up period was 21.66 ± 7.93 months (range, 12 to 26 months). At the final follow-up, the mean preoperative flexion and extension were 79.32 ± 4.52° and 74.40 ± 4.36° respectively. The mean preoperative pain score, grip strength, and Mayo wrist score were 32.48 ± 4.00; 23.88 ± 8.38 kg, and 77.72 ± 8.31 respectively. The mean postoperative flexion and extension of the wrist were 80.56 ± 6.32° and 75.43 ± 3.12° respectively. The mean postoperative pain score, grip strength, and Mayo wrist score were 12.41 ± 3.27, 26.31 ± 8.30 kg, and 90.71 ± 7.97 respectively. There were significant differences in pain, grip strength, and Mayo wrist score (P < 0.05), but no significant differences concerning the range of motion of the wrist.

**Conclusion:**

In the treatment of the nonunion of type II ulnar styloid fractures, the resection of the avulsed fragments followed by the reinsertion of the TFCC and the ulnar collateral ligament with an anchor was a reliable alternative technique, bringing the satisfactory function of the wrist.

## Introduction

Ulnar styloid fractures occur as isolated injuries or in association with distal radius and other wrist fractures [1]. Ulnar styloid fractures account for 11–33% of all distal radius and ulnar fractures [2]. The incidence of ulnar styloid nonunion is as high as 60% [3]. Although the symptomatic ulnar styloid nonunion is rare, patients are unable to move their wrists or hold objects due to wrist pain and the instability of the distal radial ulnar joint (DRUJ) [4]. The symptomatic ulnar styloid nonunion is a surgical goal to improve comfort and functions, but the optimal treatment remains controversial.

**Fig. 1:**
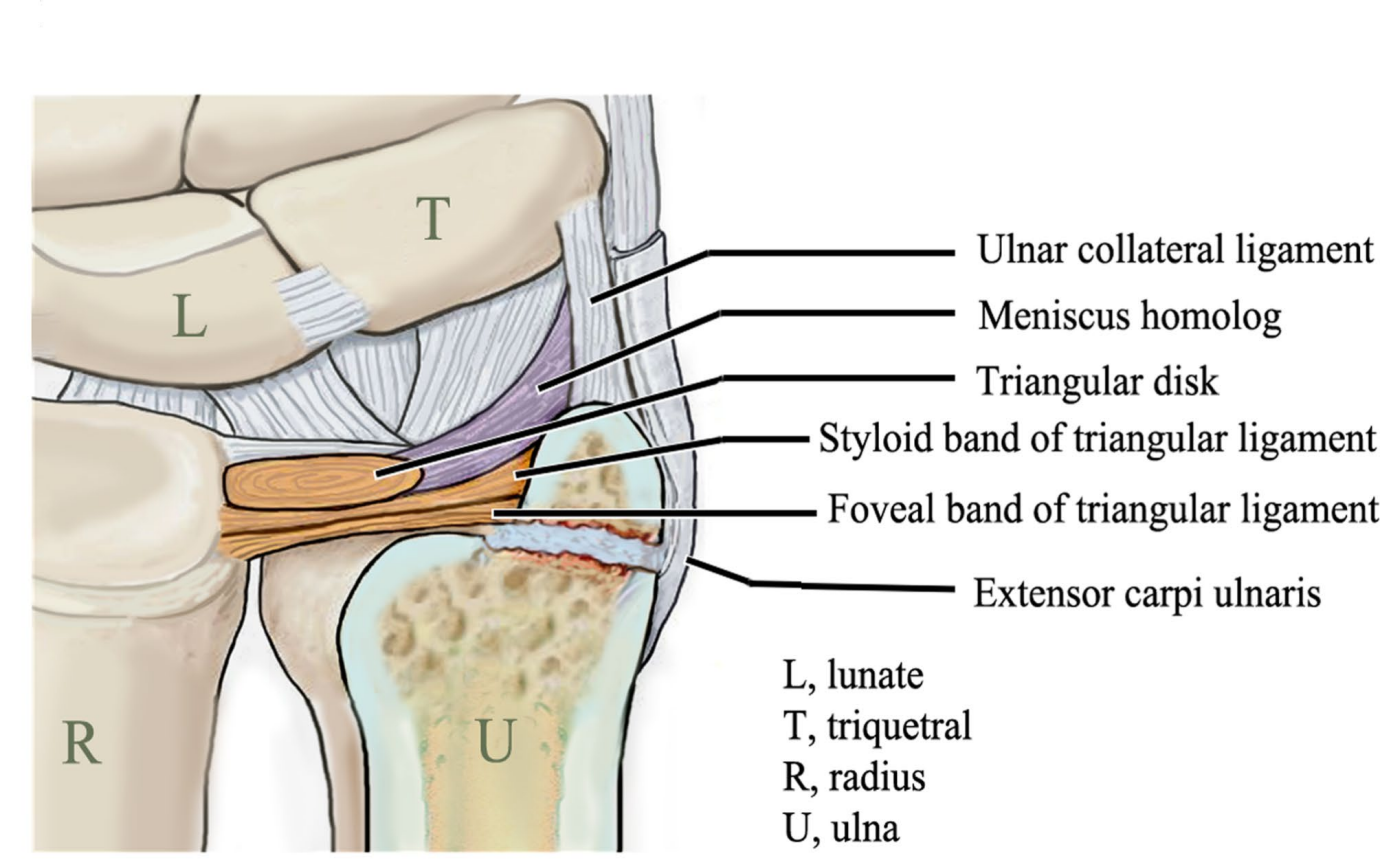
A type 2 ulnar styloid nonunion

The triangular fibrocartilage complex (TFCC) is located at the base of the ulnar styloid or fovea. The structures consist of the articular disc, the dorsal and volar radioulnar ligaments, the meniscus homologue, the ulnar collateral ligament, and the sheath of the extensor carpi ulnaris [5]. Hauck et al. [6] classified ulnar styloid nonunions into type I nonunions (located at the tip of the styloid; the TFCC remains intact; and the DRUJ is stable) and type II nonunions (occurred at the base of the styloid; and the attachment to the TFCC is disrupted, resulting in an unstable DRUJ) (Fig. 1). The ulnar styloid nonunion is usually defined as a visible fracture line on posteroanterior X-ray for at least six months after injury [7].

Simple excision of the ulnar styloid fragment may relieve wrist pain, but wrist instability is a major concern [8]. Many surgical techniques have been reported to restore the stability of the wrist. The commonly used techniques include fracture osteosynthesis using a mini plate and the screw system, tension band wiring, and a minscrew or K-wire [9]. However, the fragments are usually too small to be captured, and fracture nonunions are a major concern. Alternatively, the fragment can be excised followed by reinserting the TFCC and ulnar collateral ligament into the capsule, which avoids those drawbacks [10]. The procedures can be done arthroscopically or through open surgeries. However, the capsule is too weak to secure the TFCC and ulnar collateral ligament together, resulting in wrist instability.

The purpose of this retrospective study was to introduce an alternative technique for the treatment of type II symptomatic ulnar styloid nonunions by reinserting the TFCC and ulnar collateral ligament. We also reported the results using the technique.

## Patients and methods

This study was approved by the institutional review boards of the hospitals involved. Informed consent was obtained from each patient.

**Fig. 2:**
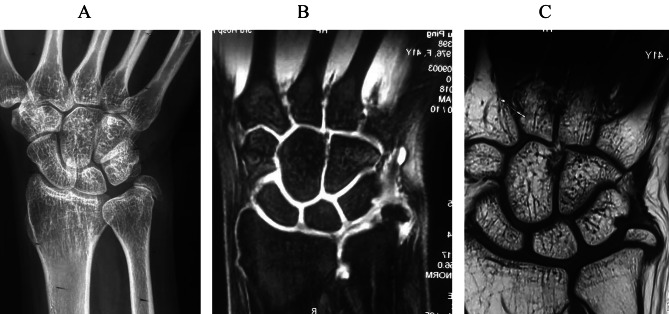
A type 2 ulnar styloid nonunion. (**A**) X-ray. (**B**) T1-weighted MRI shows a chondral defect over the triquetrum and intake of the triangular fibrocartilage complex. (**C**) T2-weighted MRI.

From March 2009 to May 2017, 63 patients with painful styloid nonunion were treated consecutively in our hospitals (Fig. 2A, B, C). Before the operation, all patients underwent X-rays, CT images, and magnetic resonance images to confirm the diagnosis. The eligibility criteria were: (1) patients aged between 18 and 65; (2) patients with a confirmed type II ulnar styloid nonunion on X-ray for at least six months after injury; (3) patients with persistent pain at the fracture site for at least six months; and (4) patients with an unstable DRUJ or not. The exclusion criteria were: (1) patients younger than 18 (because of their immature skeletons); (2) patients older than 65 (because of the osteoporosis) (n = 1); (3) patients with nonunions for less than 6 months after injury (n = 2); (4) patients with asymptomatic ulnar styloid nonunions (n = 3); (5) patients with other fractures, soft tissue injuries, or pathological fractures (n = 10); and (6) patients with diabetes, osteoarthritis, gout, and infectious diseases (n = 2). All operations were performed by the same senior surgeon.

### Surgical technique

**Fig. 3:**
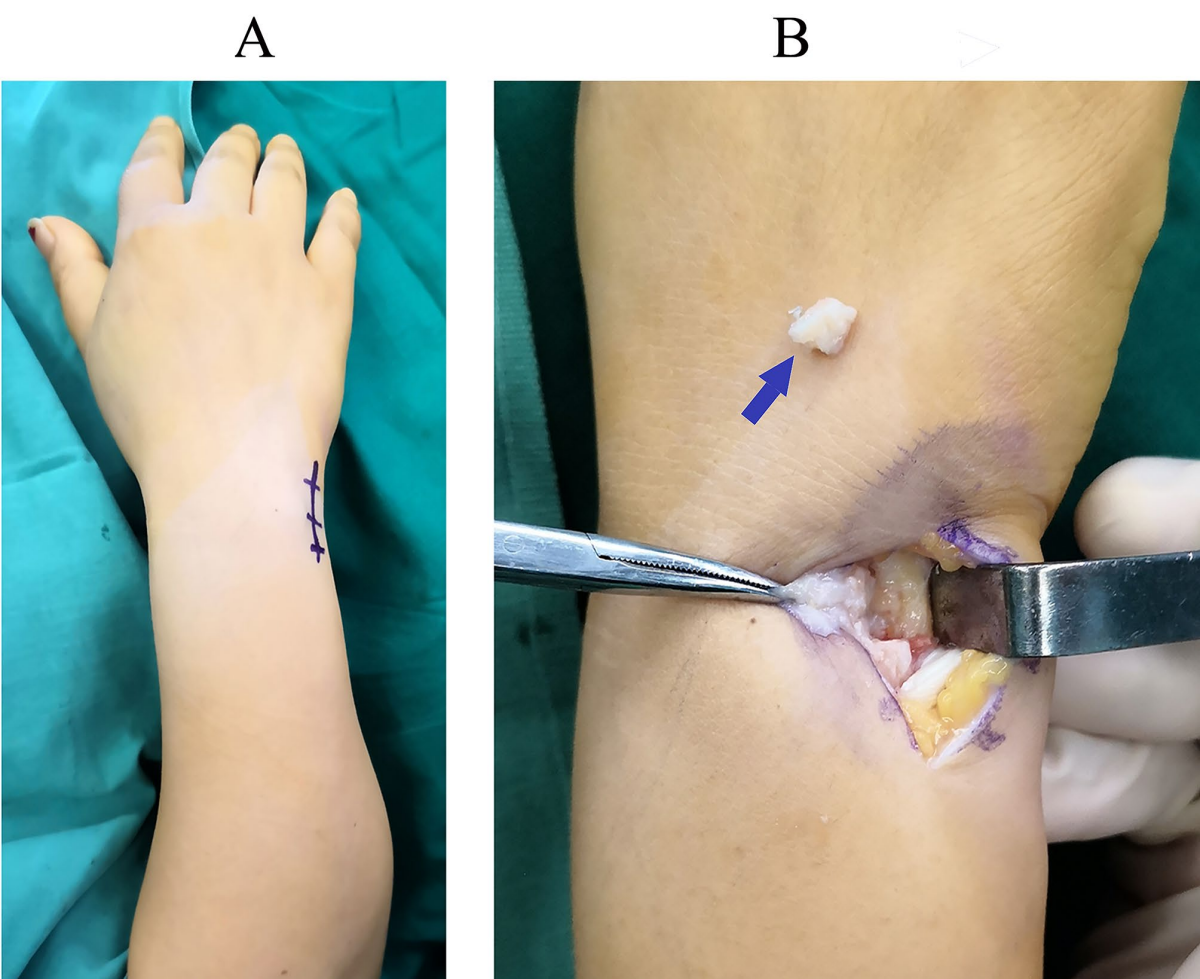
**A**. A 3-cm longitudinal incision is made over the ulnar styloid fragment. **B**. The ulnar styloid fragment (arrow) is removed

**Fig. 4:**
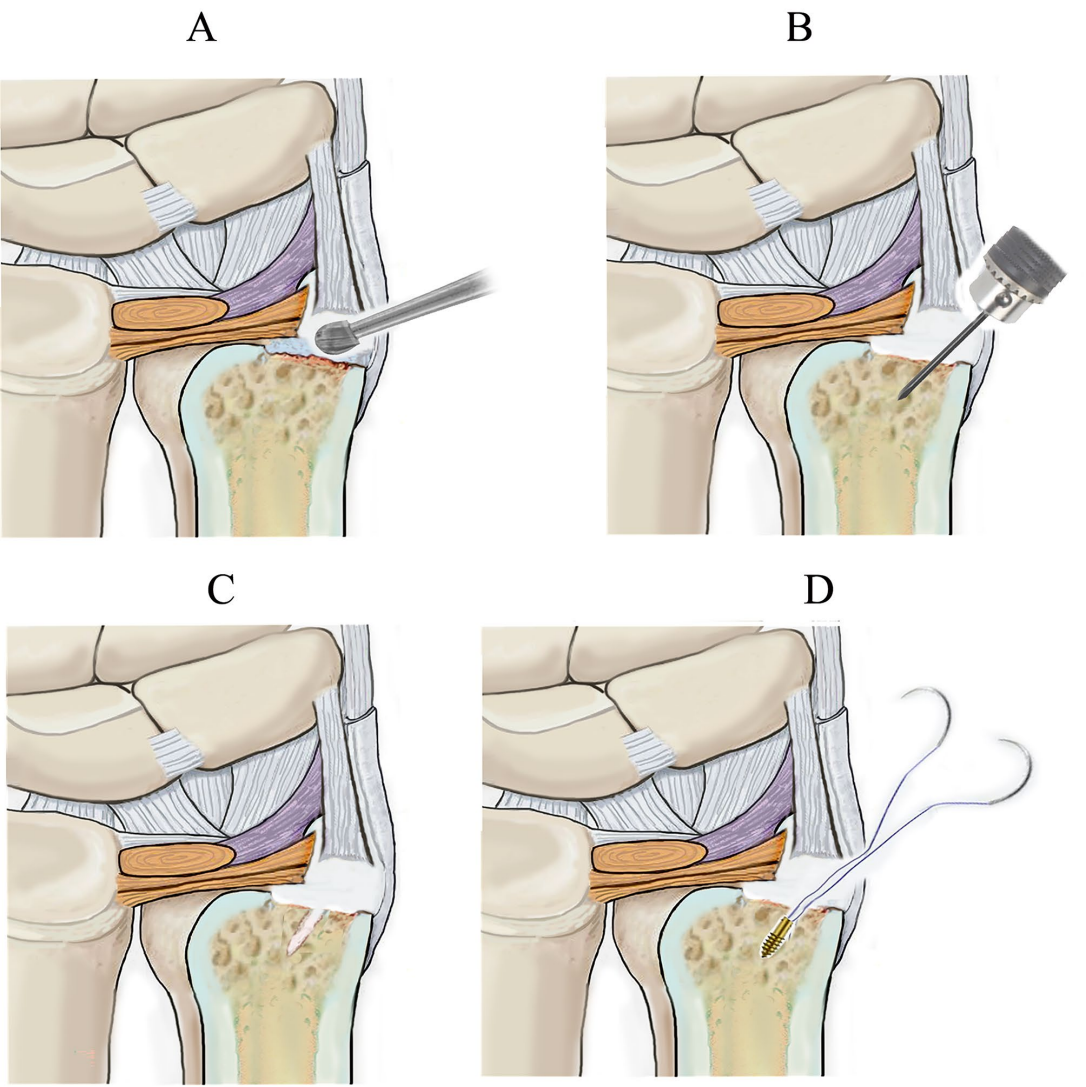
**A**. A reinsertion point is created with a bur. **B**. A drill hole is made with a 1.5 mm K-wire. **C**. The drill hole is penetrated through the first cortex. **D**. A bony tunnel is made with towel cramps

**Fig. 5:**
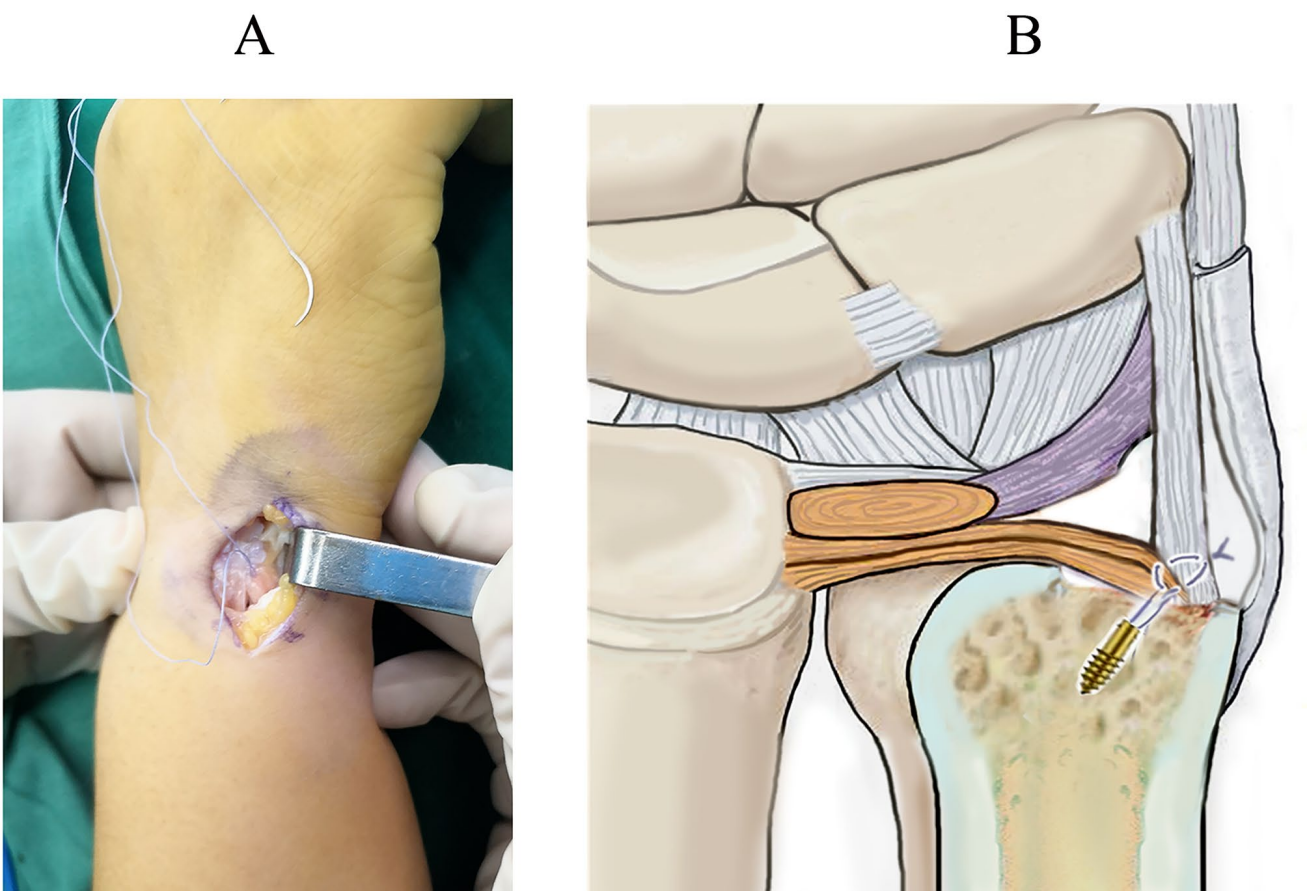
**A**. A bone anchor is driven into the ulna. **B**. The two limbs of the sutures are tied to reinsert the TFCC and ulnar collateral ligament

The operation was performed under the brachial plexus block, with the elbow flexed 90° and the forearm pronated. A pneumatic tourniquet was applied to obtain a bloodless surgical field. We made a 3-cm longitudinal incision on the dorsoulnar aspect of the distal ulna, centering around the ulnar styloid (Fig. 3A). The dorsal sensory branch of the ulnar nerve was identified and protected. We incised the dorsal retinaculum to expose the ulnar styloid process directly through the interval between the flexor carpi ulnaris and extensor carpi ulnaris tendons. The nonunion site and the avulsed fragment of the ulnar styloid process were identified. The TFCC and ulnar collateral ligament were sharply elevated from the bone fragment, and the ulnar styloid fragment was dissected subperiosteally and excised (Fig. 3B). The integrity of the superficial limb (styloid band) and deep limb (foveal band) of the TFCC and ulnar collateral ligament were carefully preserved at the insertion sites. At the base of the ulnar styloid process, the scar tissue was removed using a scalpel, and a bony insertion site was created with a bur or finger rongeur for ligament reattachment (Fig. 4A). Using a 1.5-mm K-wire, we made a drill hole penetrating the first cortex 1 cm proximal to the base of ulnar styloid process (Fig. 4B, C). Using towel clamps, we made a bony tunnel from the drill hole to the base of ulnar styloid process, where an insertion point for the ligaments was created (Fig. 4D). A 2.7 mm absorbable bone anchor (DePuy Synthes, USA) was driven into the ulna at the level about 1 cm proximal to the drill hole (Fig. 5A). We passed the two needles with two 3 − 0 FiberWire sutures through the bony tunnel. The sutures were passed through the leading edges of TFCC and ulnar collateral ligament and tightened to reduce them down to the insertion point (Fig. 5B). The two limbs of the sutures were simply tied together. After surgery, the wrist was immobilized in a dorsal splint with the forearm supinated at approximately 60°. The splint was removed six weeks after surgery, and the range of motion exercises was started thereafter.

### Outcome evaluation

**Fig. 6:**
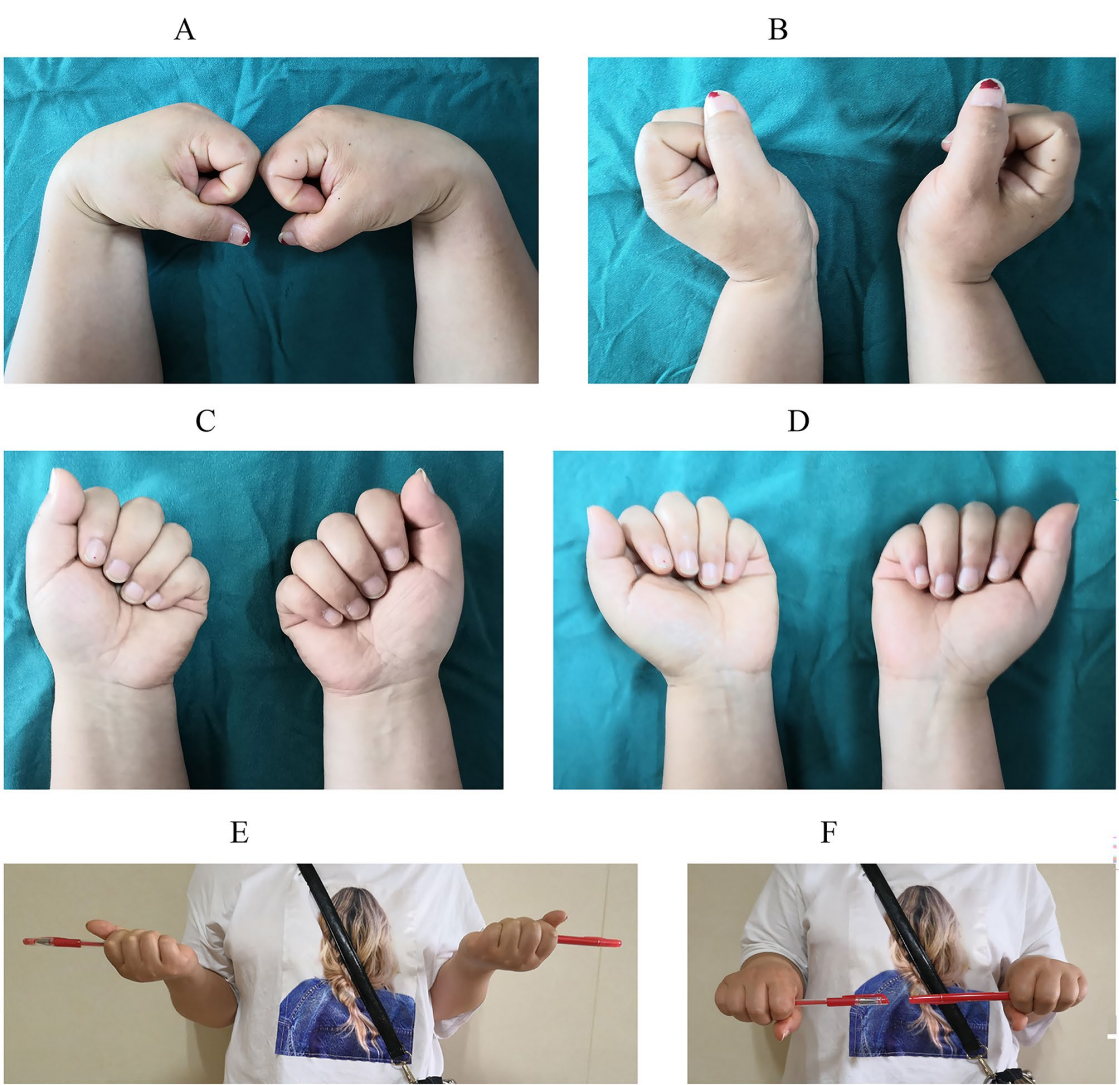
Range of motion of the right wrist after one year. (**A**) Flexion. (**B**) Extension. (**C**) Ulnar deviation. (**D**) Radial deviation. (**E**) Supination. (**F**) Pronation

Assessments included the range of motion of the wrist and the forearm rotation (Fig. 6). The grip strength of the hand was measured with a goniometer [11]. Patients rated wrist pain using the visual analog scale (0 = no pain and 100 = severe pain) [12]. We used the Mayo Wrist Score to assess the wrist function (90–100, excellent; 80–90, good; 60–80, satisfactory; below 60, poor) [13]. Hand surgeons not involved in patients’ treatment measured all parameters.

### Statistical analysis

Pre- and postoperative characteristics and outcome scores were compared. A P < 0.05 was considered statistically significant. The collected data were analysed using Statistical Package for Social Sciences v13.0 (SPSS Inc., Chicago, IL, USA).

## Results

The mean age of the 45 patients (34 males and 11 females) undergoing the surgery was 45.48 ± 11.50 years (range, 19 to 65 years) old. All patients had sustained injuries. The average time from injury to surgery was 17.57 ± 7.89 months (range, 9 to 22 months). The mean preoperative wrist pain score, grip strength, and Mayo wrist scores were 32.48 ± 4.00 mm, 23.88 ± 8.38 kg, and 65.72 ± 8.31 respectively (Table 1).

**Table 1:** Patients’ demographics and surgical details (n = 45)

Age (mean; range) (year)	45.48 ± 11.50 ; 19–65
Sex (M/F)	34:11
Side (L:R)	17:28
Cause (n)	
RTA	5
Falling	28
Machinery	6
Sports	6
TFITO (mean; range) ( month)	17.57 ± 8.89; 9–22
FP (mean; range) ( month)	21.66 ± 7.93; 19–65

RTA, road traffic accident; TFITO, time from injury to surgery; FP, follow-up period

The mean follow-up period was 21.66 ± 7.93 months (range, 12 to 26 months). At the final follow-up, the DRUJ instability was achieved in all patients. The mean postoperative pain score of the wrist, grip strength, and Mayo wrist scores were 12.41 ± 3.27; 26.31 ± 8.30 kg, and 90.71 ± 7.97 respectively (Table 2).

**Table 2:** Pre- and post-operative details and outcomes (n = 45)

Items	Preop	Postop	P value
Pain (VAS, mm)	32.48 ± 4.0	12.41 ± 3.27	0.0001
Wrist ROM (°)			
Flexion	79.32 ± 4.52	80.56 ± 6.32	0.223
Extension	74.41 ± 0.36	75.43 ± 3.12	0.238
Radial deviation	24.00 ± 4.48	23.19 ± 5.12	0.189
Ulnar deviation	37.66 ± 1.48	38.54 ± 3.48	0.063
FRA (°)	138.25 ± 4.54	137.19 ± 3.88	0.189
Grip strength (kg)	23.88 ± 8.38	26.31 ± 8.30	0.0012
Mayo wrist scores	65.72 ± 8.31	90.71 ± 7.97	0.0001
Rank (n)			
Excellent	0	29	
Good	0	14	
Fair	3	2	
Poor	42	0	

VAS, visual analogue scale; ROM, range of motion; FRA, forearm rotation arc

There were significant differences in the pain score (P = 0.0001), grip strength (P = 0.0012), and Mayo wrist scores (P = 0.0001). There were no significant differences regarding wrist flexion (P = 0.223), and wrist extension (P = 0.238). There were 29 excellent, 14 good, and 2 fair results.

## Discussion

Ulnar styloid fractures are often accompanied by radius fractures. The most common cause of an ulnar styloid fracture is a fall on an outstretched arm. Most of these are small avulsion fractures (type I) involving the tip of the ulnar styloid. The ulnar styloid is an important structure for the TFCC. Some clinical findings help establish the diagnosis of symptomatic ulnar styloid nonunion, including wrist pain on resisted ulnar deviation and dorsiflexion, a clicking sensation on wrist movement, reduced grip strength, and tenderness and swelling over the dorsal ulnar aspect of the ulna [14, 15]. Surgical treatments are indicated for symptomatic ulnar styloid nonunion.

Many surgical treatments are available for the treatment of symptomatic type II ulnar styloid nonunion, such as fixation with K-wires, tension banding wiring, compression screws, and mini-fragment plates [16, 17]. Hauck [6] treated three patients with large bone fragments with open reduction and internal fixation using tension band wiring. Two patients were treated using an AO screw. The wrist pain of the three patients was relieved after surgery, and achieved a full range of motion. Bone healing was achieved in all patients. Nunez [18] reported nonunion of the base of the ulnar styloid was treated using a plate and screw system. Bone healing was achieved in all patients, but osteosynthesis was costly. The prominence of the implant is not uncommon after the fixation of the ulnar styloid [19, 20]. The technique may not be appropriate for a small fragment. The TFCC reattachment relies on healing between the ligaments and capsule or distal ulna [21, 22], which may be suitable for a small fragment. Screw anchor backout is possible even though this complication did not happen in our series. We checked TFCC healing by testing the stability of the DRUJ. In the future, we will check the healing using MRI to better ascertain the outcome of the technique.

Our technique avoids the prominence of the implant and tendon irritation. The loss of anatomical structure and the disability of bony union are the main disadvantages. The indication of our technique is failed conservative treatments of type II ulnar styloid nonunion with persistent ulnar-sided wrist pain, especially due to small fragments.

The study has limitations. There was neither a control group nor randomization because of the small sample size.

## Conclusion

In the treatment of the nonunion of type II ulnar styloid fractures, the resection of the avulsed fragments followed by the reinsertion of the TFCC and ulnar collateral ligament with an anchor was a reliable alternative technique, bringing the satisfactory function of the wrist.

## Data Availability

All the data will be available upon motivated request to the corresponding author of the present paper.

## Abbreviations

DRUJ: Distal radial ulnar joint
TFCC: Triangular fibrocartilage complex
AO: Arbeitsgemeinschaftfür Osteosynthesefragen
CT: Computed tomography
